# 1,3-Dicaffeoylquinic Acid as an Active Compound of *Arctium lappa* Root Extract Ameliorates Depressive-Like Behavior by Regulating Hippocampal Nitric Oxide Synthesis in Ovariectomized Mice

**DOI:** 10.3390/antiox10081281

**Published:** 2021-08-12

**Authors:** Dong Wook Lim, Minji Kim, Minseok Yoon, Jaekwang Lee, Changho Lee, Min Young Um

**Affiliations:** 1 Division of Functional Food Research, Korea Food Research Institute, Wanju 55365, Korea; dwlim@kfri.re.kr (D.W.L.); msyoon@kfri.re.kr (M.Y.); jklee@kfri.re.kr (J.L.); chang@kfri.re.kr (C.L.); 2 Division of Food Biotechnology, University of Science and Technology, Daejeon 34113, Korea; 50036@kfri.re.kr

**Keywords:** *Arctium lappa* root, menopause, depression, nitric oxide, neuronal nitric oxide synthase, ERK–CREB–BDNF signaling

## Abstract

Menopause is a risk factor for depression. Although 1,3-dicaffeoylquinic acid (1,3-diCQA), a phenolic compound in *Arctium lappa* (*A. lappa*) root, has various health benefits, its effects on menopausal depression remain to be determined. Therefore, this study investigates the antidepressant-like effects of 1,3-diCQA from an *A. lappa* root extract (AE) and the associated molecular mechanisms. Ovariectomized (OVX) mice were orally administered AE for 20 weeks, following which depression-like behaviors were assessed. Although the mice exhibited depression-like behaviors, AE administration mitigated these symptoms by activating the ERK–CREB–BDNF pathway and increasing nNOS levels in the hippocampus. Similarly, a significant increase in nNOS-derived NO production and activation of the ERK–CREB–BDNF pathway was observed in the primary hippocampal neurons. Although this stimulatory effect of 1,3-diCQA was not significantly affected by treatment with estrogen receptor agonist or antagonist, it was inhibited by 7-NI, an nNOS inhibitor. Moreover, mice treated with 1,3-diCQA exhibited a marked improvement in their forced swimming test and tail suspension test immobility, while pretreatment with 7-NI reversed the antidepressant-like effects of 1,3-diCQA. Our results suggest that 1,3-diCQA regulates nNOS in an estrogen recepters-independent manner to increase NO production in OVX mice.

## 1. Introduction

Epidemiological and clinical studies have shown that women have a stronger predisposition for depression than men; this increased vulnerability has been linked to the dysregulation of the reproductive system [1]. Thus, postmenopausal women experience considerable psychological and biological changes, including major depression, due to decreased levels of estrogen [2]. Menopausal hormone therapy (MHT) has been most successfully adopted as a beneficial treatment for depression [3]. MHT encompasses several drug classes, including estrogens, progestogens, and synthetic steroids (e.g., tibolone) [4]. However, due to the serious adverse events associated with MHT, including the increased risk of breast cancer [5], alternative treatment options continue to be investigated, including traditional herbs and dietary supplements to manage postmenopausal symptoms.

Nitric oxide (NO), a free radical with diverse physiological and pathological functions, is produced by NO synthase (NOS) using L-arginine as a substrate and has been recognized as an important mediator in neuronal disorders [6]. Previous studies have indicated that NOS inhibition may have a role in the pathophysiology of depression by modulating the levels of certain neurotransmitters, including serotonin [7] and dopamine [8]. Additionally, NO has been shown to induce depression-like behaviors in an animal model via modulating the cyclic guanosine monophosphate (cGMP) [9]. However, contrary to these studies, the level of neuronal NO synthase (nNOS) has been found to be significantly lower in the locus coeruleus region of the brain in patients suffering from major depression compared to normal healthy subjects [10]. Meanwhile, MHT reportedly causes a significant increase in plasma NO levels and improves menopausal psychological symptoms, including depressive moods and sleep disturbances [11]. Moreover, 17β-estradiol (E2) induces hippocampal nNOS expression via activation of estrogen receptors (ERs), a phenomenon that subsequently induces phosphorylation of cAMP response element-binding protein (CREB) in hippocampal neurons [12]. Meanwhile, hippocampal NO is believed to be essential for the behavioral effects of E2, whereas nNOS is necessary for ERβ-mediated CREB activation in the female hippocampus. Hence, NO depletion results in depression-like behaviors by affecting CREB activation [13]. As such, although the role of NO in the pathophysiology of depression remains obscure [14], we postulate that the nNOS-CREB signaling pathway has an important role in postmenopausal depression.

*Arctium lappa*, commonly known as burdock, is a traditional Chinese medicinal herb and an edible perennial plant belonging to the Asteraceae family [15]. Previous studies have reported burdock to exhibit antioxidant properties [16,17,18]. Several studies have also reported that *A. lappa* root has various pharmacological activities, including anti-inflammatory [19], antibacterial [20], hepatoprotective [21], gastroprotective [22], and neuroprotective activities [23]. Specifically, the root of *A. lappa* is rich in polyphenol compounds, containing mono- and di-caffeoylquinic acid as the major chemical components [24]. Polyphenols have a high antioxidant capacity and have demonstrated beneficial effects in many oxidative-stress-related diseases, including depression [25]. Moreover, *A. lappa* root extract, and its active compounds, have been shown to elicit protective effects toward neuronal atrophy and alleviate stress-hormone-induced depressive behaviors in mice [26]. However, the antidepressant-like effects of *A. lappa* root extract, and its active component, 1,3-dicaffeoylquinic acid (1,3-diCQA), in depressive mood-related menopause have not yet been defined.

Therefore, in the current study, we sought to examine the efficacy of 1,3-diCQA as an active compound of *A. lappa* root extract (AE) in ovariectomized (OVX) mice with depression induced by estrogen deficiency. We further elucidated the molecular mechanisms of action responsible for the effect of AE on NO production and the estrogen deficiency-related ERK–CREB–BDNF signaling network.

## 2. Materials and Methods

### 2.1. Reagents

1,3-diCQA, poly L-omithine hydobromide, 0.3% Triton X-100, histology mounting medium Fluoroshield™ with 4,6-diamidino-2-phenylindole (DAPI), dimethyl sulfoxide (DMSO), donkey serum, and RIPA buffer with protease and phosphatase inhibitor cocktail were purchased from Sigma-Aldrich (St. Louis, MO, USA). 7-Nitroindazole (7-NI), DPN, ICI 182,780, and PPT were obtained from Tocris Bioscience (Ellisville, Missouri, USA). Dulbecco’s modified Eagle’s medium (DMEM), fetal bovine serum (FBS), phosphate-buffered saline (PBS), 2.5% trypsin-EDTA, neurobasal medium A, B-27, L-glutamine, and penicillin/streptomycin were obtained from Gibco (Grand Island, NY, USA). Trypsin-EDTA (0.25%) was obtained from Hyclone (Logan, UT, USA). Hanks’ balanced salt solution (HBSS) was purchased from Welgene Inc. (Gyeongsan, South Korea). 4-amino-5-methylamino-2′,7′-difluorofluorescein diacetate (DAF-FM diacetate) fluorescence was obtained from Invitrogen (Carlsbad, CA, USA). The Griess assay reagent kit was supplied by Promega Biotech Co. (Madison, WI, USA). Goat serum was obtained from Vector Laboratories (Burlingame, CA, USA). The BCA protein kit was purchased from Thermo Fisher Scientific Inc. (Waltham, MA, USA). Tris-buffered saline (TBS) and chemiluminescent reagent were obtained from Santa Cruz Biotechnology (Santa Cruz, CA, USA). Nucleospin^®^ RNA XS kit was purchased from Macherey-Nagel (Düren, Germany). ReverTra Ace qPCR RT Master Mix kit was obtained from TOYOBO (Osaka, Japan), and SYBR Green Real-time PCR Master Mix was obtained from Takara (Kyoto, Japan). Imipramine hydrochloride was purchased from Wako Pure Chemical Corporation (Tokyo, Japan).

### 2.2. Sample Preparation and HPLC Analysis

**Figure 1:**
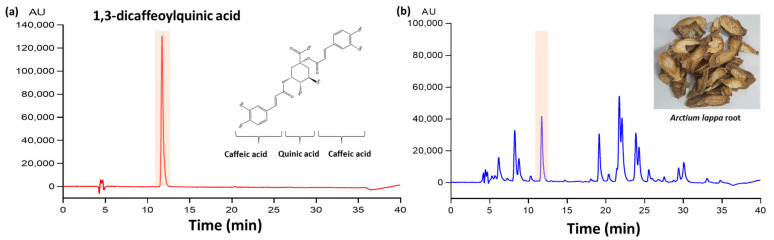
HPLC chromatogram of (**a**) 1,3-dicaffeoylquinic acid (1,3-diCQA) as standard compound and (**b**) *Arctium lappa* root extract (AE). The concentration of 1,3-diCQA was 1.35 ± 0.02 mg/g AE.

The dried roots of *A. lappa* were purchased from BUYOUNG Farm Inc. (Andong, South Korea), and the root (100 g) was extracted in 20% ethanol (1000 mL, *v*/*v*) using a reflux apparatus at 70 °C for 6 h. After removing the solvents via rotary evaporation, the extract was freeze-dried at a 45% yield (*w*/*w*). The sample was dissolved in 50% methanol and analyzed using the HPLC system (Jasco, Tokyo, Japan) with Waters Symmetry ^®^ C18 column (5 μm, 4.6 × 250 mm). The mobile phase was composed of 0.2% formic acid (A) and MeOH (B). The separations were processed with a solvent (A) gradient that decreased from 85% to 10% within 35 min, and then increased to 85%, with a 40 min running time and an injection volume of 10 μL. The flow rate was 0.8 mL/min, and sample was detected at 330 nm. The regression equation and correlation coefficient (R2) of each standard curve were automatically determined using HPLC system. The regression equations for 1,3-diCQA were y = 3617.3x + 3571.3 (R^2^, 0.9995). HPLC quantitative analysis was replicated three times. The concentration of 1,3-diCQA was 1.35 ± 0.02 (mean ± SD) mg/g extract using peak area in the standard chromatogram. A representative chromatogram of the 1,3-diCQA reference and its corresponding peak in the AE is illustrated in Figure 1.

### 2.3. Animals

All animal experiments were approved by the Institutional Animal Care and Use Committee of the Korea Food Research Institute (IACUC number, KFRI-M-19037). Female C57BL/6N mice (5 weeks old, weighing 15–20 g) were purchased from KOATECH Animal Inc. (Pyeongtaek, South Korea) and were housed in cages (4–5 mice per cage). Mice were provided with ad libitum access to food and water under a controlled temperature (21 ± 2 °C) with a 24 h (12 h:12 h) light–dark cycle: lights on at 07:00 and lights off at 19:00. The mice were acclimated for a minimum of 1 week prior to experimentation.

### 2.4. Ovariectomy and Treatments

After the 1-week acclimatization period, female C57BL/6N mice were anesthetized with 2% isoflurane. Both ovaries were ovariectomized (OVX) via small bilateral dorsal flank incisions and subsequent removal of ovaries at 6 weeks of age. SHAM-operated mice received similar incisions without removal of the ovaries.

#### 2.4.1. Experiment 1

**Figure 2:**
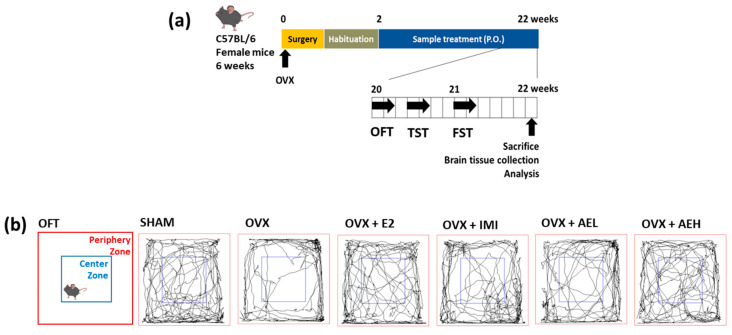

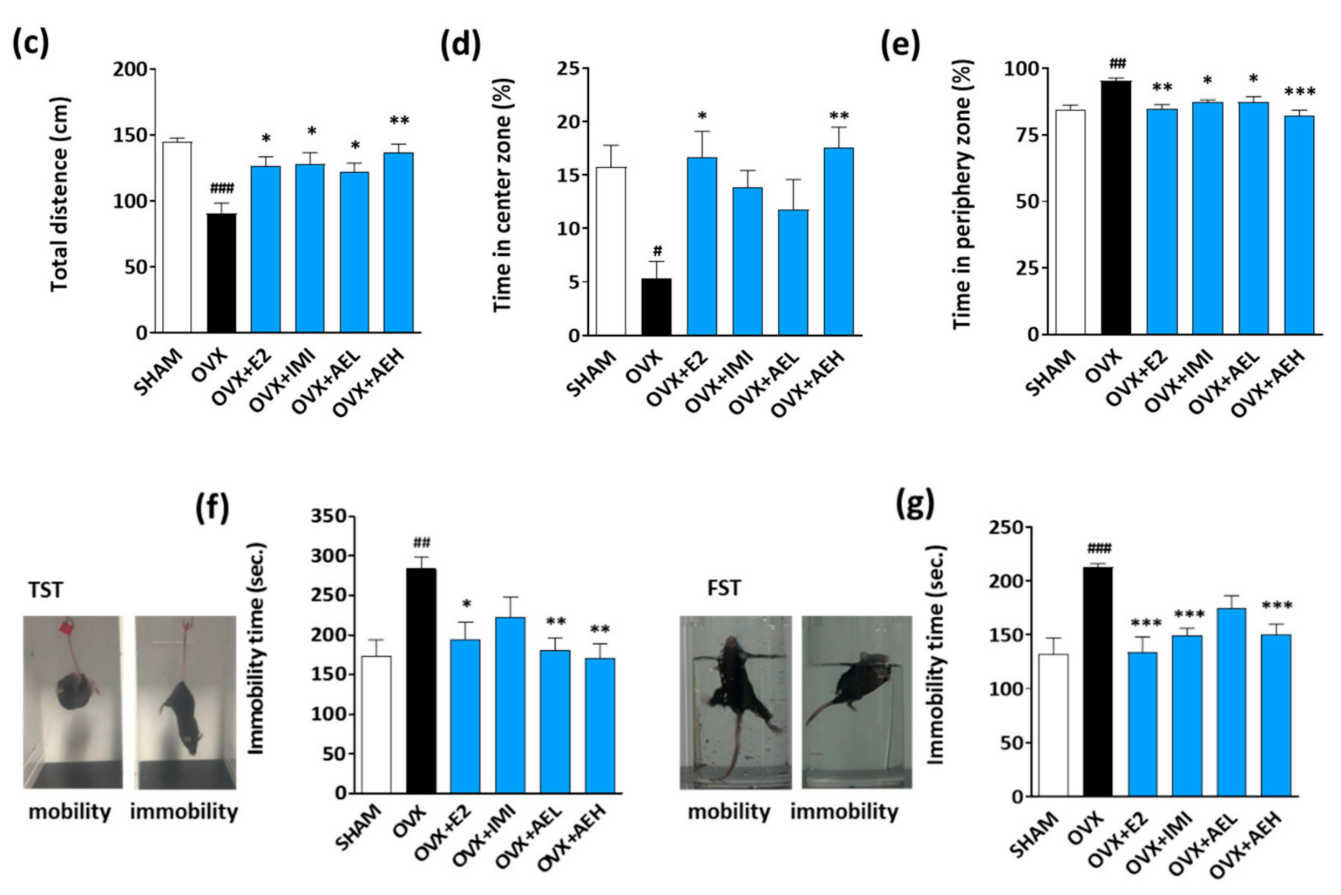
AE improves depressive-like behavior in OVX mice. (**a**) Experimental design and treatment schedule. OVX mice were orally administered AE (100 and 300 mg/kg, p.o.) for 20 weeks. (**b**) The tracing of the locomotor activity for 5 min. OVX mice exhibited depressive-like behavior, while AE led to significantly improved behaviors; (**c**) total distance, (**d**) time in center zone, and (**e**) time in periphery zone. OVX mice exhibited significantly increased immobility, while AE-treated mice showed significant improvements, with a reduced immobility time in the (**f**) TST and (**g**) FST. Results are presented as mean ± SD. OFT, open filed test; TST, tail suspension test; FST, forced swim test; E2, 17β-estradiol 10 µg/kg; IMI, imipramine 30 mg/kg; AEL, AE 100 mg/kg; AEH, AE 300 mg/kg. ^#^ *p*  <  0.05, ^##^ *p* <  0.01, and ^###^ *p* < 0.001 vs. SHAM group; * *p* < 0.05, ** *p* < 0.01, *** *p* < 0.001 vs. OVX group.

After a 2-week recovery period from the OVX surgery, C57BL/6N female mice (8-weeks old) were classified into the following six experimental groups: (1) SHAM; (2) OVX-control (OVX); (3) 17β-estradiol 10 µg/kg (OVX+E2); (4) imipramine 30 mg/kg (OVX+IMI); (5) AE 100 mg/kg (OVX+AEL); and (6) AE 300 mg/kg (OVX+AEH)-treated groups (n = 8 mice per group). The dose of AE in the current study was selected from previous reports [26]. AE at a dosage of 300 mg/kg in mice corresponds to 1.45 g/60 kg human, where AE is extracted from approximately 3.22 g of the *A. lappa* root raw material; this dosage is similar to dosage of *A. lappa* root in drinking or supplementation in traditional Chinese medicine [27]. IMI and AE were orally administered following dissolution in distilled water at the desired doses in a volume of 10 mL/kg. E2 was dissolved in physiological saline with 1% DMSO and 1% Tween 80 and was administered via intraperitoneal (i.p.) injections. All groups were treated once daily for 20 weeks. After 18 weeks of treatment, behavior tests were initiated 1 h after sample treatment, according to the experimental scheme presented in Figure 2a. Immediately after sacrifice, brain tissues were isolated and stored at −80 °C until analysis.

#### 2.4.2. Experiment 2

OVX mice were divided into four groups (n = 8 mice per group) as follows: (1) Group 1, OVX animals receiving 0.9% saline (p.o.) and vehicle (i.p.); (2) Group 2, OVX animals receiving 1,3-diCQA 30 mg/day (p.o.) and vehicle (i.p.); (3) Group 3, OVX animals receiving 0.9% saline (p.o.) and 7-NI (selective nNOS inhibitor, i.p.); (4) Group 4, OVX animals receiving 1,3-diCQA 30 mg/day (p.o.) and 7-NI (i.p.). To determine relevant dose of 1,3-diCQA in the current study, we translated the doses for animals from human studies according to previous [28]. To convert the dose used in a mouse to a dose based on surface area for humans, multiply 30 mg/kg by the Km factor, 3 for a mouse, and then divide by the Km factor, 37 for a human. This calculation results in a human equivalent dose for 1,3-diCQA of 2.44 mg/kg, which equates to a 146.34 mg dose of 1,3-diCQA for a 60 kg person. The OVX mice were administered i.p. injection of 7-NI (50 mg/kg) once daily for 5 days, while those in the untreated 7-NI groups were injected with an equal volume of vehicle (0.9% saline with 1% DMSO and 1% Tween 80). On day 5, behavior analysis was initiated 1 h after treatment was administered, according to the experimental scheme presented in Figure 7.

### 2.5. Behavior Tests

#### 2.5.1. Open Field Test (OFT)

The mice were tested in an open field, and their movements were measured. The OFT was performed according to the protocol previously described [29]. Specifically, the duration of time spent in the center and periphery zone, as well as the total movement, were recorded for 5 min. The mice’s locomotor-related activity was analyzed using SMART version 3.0 software (Panlab SL, Barcelona, Spain).

#### 2.5.2. Tail Suspension Test (TST)

The TST was conducted, as previously described [30]. Immobility is perceived as abandonment behavior that can be expressed as depression in mice by TST and FST [31]. The mice were hung by their tails using adhesive tape and attached to a hook. Immobility time was evaluated in the 6 min using an automated device (BioSeb, Chaville, France).

#### 2.5.3. Forced Swimming Test (FST)

The FST is one of the most commonly used tests for the study of depressive-like behavior in rodents. To verify whether AE and its constituent had antidepressant-like effects, FST was performed as previously described [32]. The mice were placed in a clear cylinder (height; 13 cm, diameter; 24 cm) with water (depth; 10 cm, 22–24 °C) for 6 min. The immobility and swimming times were analyzed during the last 4 min using SMART version 3.0 software (Panlab SL, Barcelona, Spain).

### 2.6. Cell Culture

#### 2.6.1. SH-SY5Y Cell Culture

SH-SY5Y human neuroblastoma cells were obtained from the American Type Culture Collection (Rockville, MD, USA). Cells were cultured in DMEM supplemented with 10% FBS and 100 U/mL penicillin/streptomycin in a humidified incubator maintained at 37 °C with 5% CO_2_ and 95% air. The medium was replaced every 2–3 days, and cells were sub-cultured using 0.25% Trypsin-EDTA solution.

#### 2.6.2. Primary Hippocampal Neuron Culture

Primary hippocampal neurons were isolated and cultured with protocol (IACUC number, KFRI-M-20006). The hippocampi of postnatal day 0 (P0) ICR mice were dissected and placed in HBSS solution without Ca^2+^ or Mg^2+^ containing 1 mM sodium pyruvate and 10 mM HEPES. The tissues were incubated and inverted in HBSS solution with 2.5% trypsin for 5 min. Tissues were then triturated by repeated pipetting. The dispersed tissues were collected and centrifuged at 1600 rpm for 5 min. The pellets were then re-suspended in a neuron culture medium (Neurobasal medium A) containing B-27, 0.5 mM L-glutamine, 20 IU/mL penicillin, and 20 IU/mL streptomycin. Plates were coated with poly-L-ornithine (5 mg/mL), and cells were then plated for each experiment. Cells were incubated at 37 °C in a humidified atmosphere of 95% air and 5% CO_2_. Half of the media was replaced with fresh medium every 3 days.

For Western blot analysis, primary hippocampal cells were seeded into 6-well plates at 4 × 10^6^ cells per well. After 7 days, cells were treated with 10 nM E2 and 1 or 5 μM 1,3-diCQA for 24 h. In addition, to perform immunofluorescence analysis, cells were seeded into 12-well plates with cover glasses at 1 × 10^5^ cells per well. After 14 days, cells were treated with 10 nM E2 and 1 or 5 μM 1,3-diCQA for 24 h. To investigate the effects of 1,3-diCQA on NO production, cells were seeded into 96-well plates at 1 × 10^4^ cells per well. After 14 days, cells were treated with 10 nM E2 and 1 or 5 μM 1,3-diCQA. After 24 h, the NO content was measured.

### 2.7. Measurement of NO Contents

The level of intracellular NO was measured with an NO-specific indicator, DAF-FM. DAF-FM was dissolved in DMSO at a concentration of 5 mM and stored at −20 °C. Primary hippocampal neurons that had been treated with samples for 24 h were washed twice with PBS, after which phenol red-free DMEM was added with DAF-FM (10 μM) for 30 min at 37 °C. After washing with PBS, cells were incubated for an additional 30 min. DAF-FM fluorescence was measured using a fluorescence microplate reader at excitation wavelengths of 495 nm and emission wavelengths of 515 nm. Images of NO were captured using fluorescence microscopy (Zeiss, Thornwood, NY, USA), and NO-positive cells were counted.

In whole brain tissue, NO content was determined using a Griess assay reagent kit. Briefly, brain tissues were homogenized in PBS, centrifuged at 13,000 rpm for 10 min, and the supernatant was collected. The supernatant, sulfanilamide solution, and N-1-napthylethylenediamine dihydrochloride solution (1:1:1) were added to 1.5 mL microtubes. The mixture was centrifuged at 3000 rpm for 5 min, and the supernatant (150 μL) was added to a 96-well plate. Absorbance was then measured at 570 nm with a microplate reader (Molecular Device, Sunnyvale, CA, USA). Nitrite concentrations were quantified by the amount of protein.

### 2.8. Immunofluorescence Staining and Imaging

Primary hippocampal neurons were seeded into 12-well plates with cover glasses at 1 × 10^5^ cells per well. After 14 days, cells were treated with 10 nM E2 and 1 or 5 μM 1,3-diCQA. Immunostaining was performed as previously described [33]. Briefly, the primary hippocampal neurons were washed with 0.1 M PBS thrice and fixed with 4% paraformaldehyde in PBS for 30 min. The cells were then washed thrice with PBS and blocked with 1% goat and donkey serum and 0.3% Tritonx-100 in PBS for 1 h at room temperature (RT). The neurons were then incubated with anti-nNOS antibody (Table 1) at RT for 2 h. Next, the cells were washed thrice with PBS and incubated with Alexa Fluor-conjugated 488 and 555 secondary antibodies (1:500) for 1 h at RT. The coverslips were washed thrice with PBS and mounted on slides using a mounting solution containing DAPI. For capturing images, the Leica TCS SP8 confocal microscope (Leica Microsystems, Mannheim, Germany) was used.

### 2.9. Immunohistochemistry

After the animals were anesthetized, whole brains were removed. The brains were fixed overnight in 10% formalin and dehydrated in 30% sucrose in PBS. Brain tissues were frozen in optimal cutting temperature compound at −20 °C. Frozen brains were cut into 40 μm serial hippocampal sections on a cryostat (Leica Microsystems, Mannheim, Germany). For nNOS immunofluorescence, sections were first rinsed with PBST five times for 3 min each. The sections were subsequently blocked with 10% goat serum in PBST for 1 h at RT followed by incubation with the primary rabbit anti-nNOS antibody (1:1000) in blocking solution overnight at 4 °C. After washing with PBS five times for 3 min each, the sections were incubated with secondary antibody Alexa-flour 488 (1:500) for 2 h at RT. The sections were washed five times for 3 min each and mounted on slides using a mounting solution containing DAPI. The images were visualized using an Eclipse Ti confocal fluorescent microscope (Nikon, Tokyo, Japan).

### 2.10. Immunoblotting

Hippocampal tissues were homogenized in RIPA buffer with a protease and phosphatase inhibitor cocktail. Cell lysates were centrifuged at 13,000 rpm for 10 min at 4 °C, and the supernatants were collected. The protein concentration was determined using a BCA Protein Assay Kit. Protein samples (20 μg) were loaded and electrophoresed by 8–10% SDS-PAGE at 80 V for 120 min. The separated proteins were transferred to PVDF membranes at 1.0 A for 30 min. For blocking, the membranes were incubated with 5% nonfat dry milk in TBS containing 0.1% Tween-20 (TBST) for 1 h at RT. After washing with TBST, the membrane was incubated with primary antibodies at 4 °C overnight. The membrane was then washed with TBST thrice and incubated with HRP-conjugated secondary antibody (Santa Cruz Biotechnology, Santa Cruz, CA, USA) for 1 h at RT. After washing with TBST thrice, the membranes were detected using the ECL Western blotting detection kit (Bio-Rad, Mississauga, ON, Canada). For protein normalization, β-actin was used as a control. The relative intensities of each band were analyzed using ImageJ software (National Institutes of Health, Bethesda, MD, USA).

### 2.11. Real-Time Quantitative Polymerase Chain Reaction (RT-qPCR)

Total RNA was extracted using the Nucleospin^®^ RNA XS kit. RNA was quantified using the NanoDrop ONE (Thermo Scientific, Waltham, MA, USA). cDNA was synthesized using a ReverTra Ace qPCR RT Master Mix kit (TOYOBO, Osaka, Japan) according to the manufacturer’s protocol. RT-qPCR was performed using the QuantStudio 6 real-time PCR system (Applied Biosystems, Waltham, Massachusetts, USA) and SYBR Premix Taq in a final volume of 20 μL. The primer sequences used were as follows: mouse nNOS–forward primer: 5′-CCCACCAAAGCTGTCGATCT-3′ and reverse primer: 5′-GGAGGTTGGCCTTGGTATTT-3′; mouse β-actin forward primer: 5′-CGAGCACAGCTTCTTTGCAGC-3′ and reverse primer: 5′-CCTTCTGACCCATTCCCACC-3′. For normalization, β-actin or Gapdh were used as a housekeeping gene parallel to each gene examined.

### 2.12. Statistical Analysis

Data are presented as mean ± standard deviation (SD) and analyzed using one-way analysis of variance (ANOVA), followed by post hoc Tukey’s test using Prism 8 (GraphPad Software, Inc., San Diego, CA, USA). The threshold for significance was set at *p* < 0.05.

## 3. Results

### 3.1. Effect of AE on Depressant-Like Behaviors Caused by Estrogen Deficiency in OVX Mice

To determine whether AE alters depressive-like behavior induced by estrogen deficiency, we compared the immobility exhibited by mice during the TST and FST, as well as the total distance (cm) traveled and total time spent in the center and periphery zones during the OFT.

As shown in Figure 2b, OVX mice exhibited lower locomotor activities such as a decrease in the total distance (cm) travel and time spent in the center zone (%) and a longer period in the periphery zone (%) compared with the SHAM group. As expected, the E2 and IMI groups showed significantly improved estrogen deficiency-induced depressive-like behaviors compared to the OVX group. Similarly, AEH administration induced significant behavioral alterations in OVX mice, as evidence by a significant increase in total distance (Figure 2c) and time in the center zone (Figure 2d), as well as a significant decrease in time in the periphery zone (Figure 2e). In the TST and FST, results show that the OVX group had significantly increased immobility times compared to the SHAM group. However, AE-treated OVX mice had significantly reduced immobility times, with a maximum decrease in immobility at a dose of 300 mg/kg in the TST (Figure 2f) and FST (Figure 2g). Cumulatively, these results suggest that depressive-like behavior is induced by estrogen deficiency, whereas AE attenuates depressive-like behaviors.

### 3.2. AE Restores NO Shortage via Regulation of Hippocampal nNOS Expression in OVX Mice

**Figure 3:**
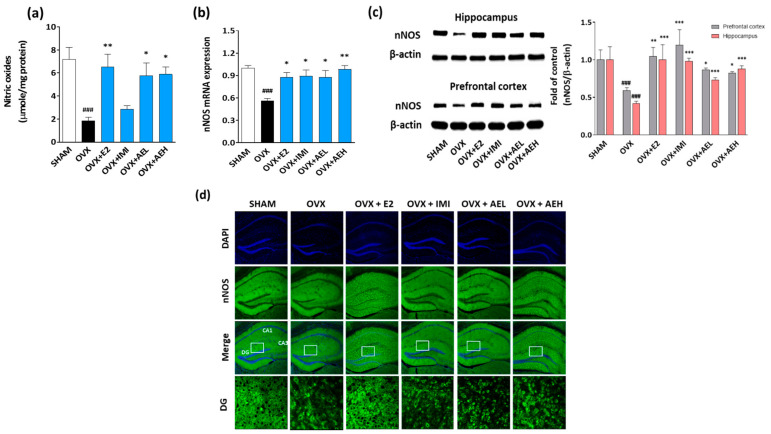
AE upregulates the hippocampal NO-mediated signaling network in estrogen deficiency-induced depressive mice. OVX mice were exposed to estrogen deficiency for 20 weeks with or without AE (100 or 300 mg/kg). (**a**) NO content in the brain tissue of OVX mice. (**b**) nNOS mRNA expression levels in the hippocampus of OVX mice. (**c**) Immunoblots showing nNOS protein levels in the hippocampus and prefrontal cortex of OVX mice. (**d**) Representative images of nNOS+ neurons from the hippocampus and DG areas. Nuclei were counterstained with DAPI (blue) and nNOS staining of hippocampus (green) X100. Results are presented as mean ± SD. ### *p* < 0.001 vs. SHAM group; * *p* < 0.05, ** *p* < 0.01, *** *p* < 0.001 vs. OVX group.

Estrogen deficiency-induced depression is related to alteration of NO production in the brain. In female mice, NO shortage in the brain causes depressive-like behaviors [34]. Based on the above-mentioned results, we hypothesized that AE could regulate NO production in the brain of OVX mice. In our results, OVX mice exhibited that NO concentration and nNOS mRNA expression was significantly lower compared to the SHAM group, while AE administration reversed this estrogen deficiency-reduced NO production in the brain of OVX mice (Figure 3a,b).

Since the hippocampus plays an important role in the pathogenesis of depression [35], we examined nNOS expressions in the hippocampus of OVX mice. As expected, the level of nNOS in the hippocampus was also significantly decreased in OVX mice, while the level of nNOS was significantly higher in OVX mice receiving AE treatment (Figure 3c). Additionally, immunohistochemistry results showed that the level of nNOS in the AE-treated groups was higher than that of the control group in the hippocampus, especially in the dentate gyrus (DG) region (Figure 3d). These results indicated that AE treatment restored the estrogen deficiency-induced NO shortage by upregulating hippocampal nNOS levels in OVX mice.

### 3.3. AE Restores Hippocampal ERK–CREB–BDNF Signaling Networks in OVX Mice

**Figure 4:**
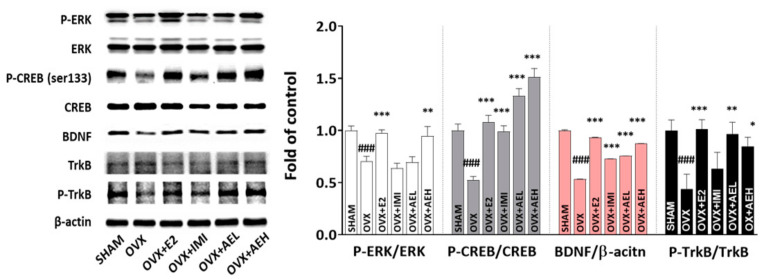
Immunoblots showing the hippocampal ERK–CREB–BDNF signaling-pathway-related protein expression levels of the OVX mice treated with AE (100 or 300 mg/kg/d, p.o.). Results are presented as mean ± SD. ### *p* < 0.001 vs. SHAM group; * *p* < 0.05, ** *p* < 0.01, *** *p* < 0.001 vs. OVX group.

The extracellular-regulated kinase (ERK)–cyclic AMP-response element-binding protein (CREB)–brain-derived neurotrophic factor (BDNF) signaling pathway is an important factor in the pathogenesis of depression [36]. Therefore, we investigated the ERK–CREB–BDNF signaling networks to determine the molecular mechanisms underlying the effects of AE in the hippocampus. As expected, the phosphorylation of ERK, CREB, and tropomyosin receptor kinase B (TrkB) receptors was significantly decreased in the OVX group compared to the SHAM group. However, these alterations were significantly rescued by AE administration, which also effectively increased BDNF protein expression levels (Figure 4). These results suggest that AE increased the levels of BDNF and phosphorylated ERK, CREB, and TrkB receptors in the hippocampus of OVX mice.

### 3.4. 1,3-diCQA Restores NO Shortage via Regulation of nNOS Expression and ERK–CREB–BDNF Signaling Networks in Primary Hippocampal Neurons

**Figure 5:**
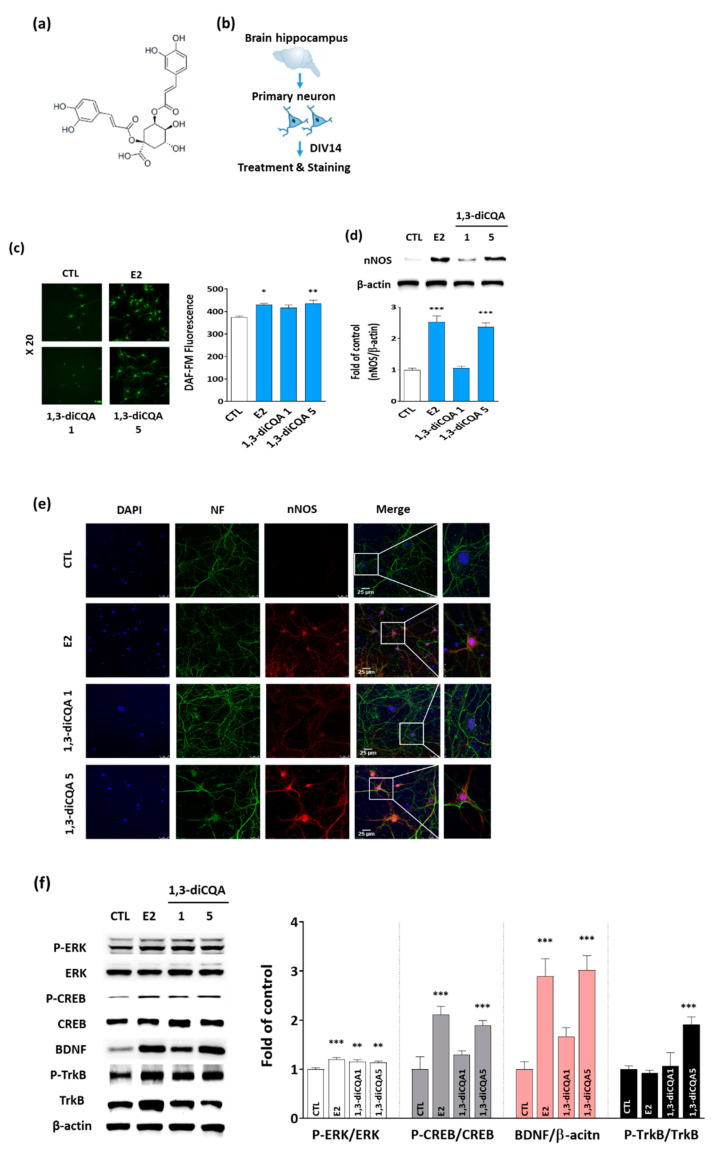
1,3-diCQA as an active compound from AE upregulates nNOS expression and the ERK–CREB–BDNF signaling pathway in primary hippocampal neuron. (**a**) Structure of 1,3-diCQA. (**b**) Schematic of primary hippocampal neuron culture and treatment. (**c**) Cultured hippocampal neuronal cells stained with fluorescent NO indicator DAF-FM DA for 30 min. NO abundance was detected via fluorescence intensity at an excitation wavelength of 495 nm and emission wavelength of 515 nm. (**d**) Immunoblots showing nNOS protein levels in neuronal cells. (**e**) Immunofluorescence analysis using primary antibodies to nNOS (red) and a neuronal marker neurofilament (NF, green). DAPI is used to identify nuclei (blue). The merged confocal image indicates that many of the 1,3-diCQA-treated cells are positive for nNOS, which is similar to results obtained following E2 treatment. Magnification, ×630. (**f**) Immunoblots showing ERK–CREB–BDNF signaling-pathway-related protein expression in hippocampal neurons treated with E2 or 1,3-diCQA. Results are presented as mean ± SD. * *p* < 0.05, ** *p* < 0.01, *** *p* < 0.001 vs. vehicle-only treated cells (CTL).

We examined the effect of 1,3-diCQA (Figure 5a) on NO production and nNOS expression in primary hippocampal neurons (Figure 5b). NO production was measured in primary hippocampal neurons exposed to 1,3-diCQA (1 and 5 μM) for 24 h using fluorescent NO indicator DAF-FM diacetate. As shown in Figure 5c, 1,3-diCQA significantly increased DAF-FM fluorescence compared with the vehicle. In addition, immunoblotting and immunofluorescence data showed an increase in the expression of nNOS in the primary hippocampal neurons of the 1,3-diCQA-treated group (Figure 5d,e).

To further investigate the involvement of the ERK–CREB–BDNF signaling network, we quantified the expression and phosphorylation levels of ERK, CREB, BDNF, and TrkB receptors in primary hippocampal neurons by immunoblotting. 1,3-diCQA treatment increased the phosphorylation of ERK, CREB, and TrkB receptors and elevated BDNF levels (Figure 5f). Taken together, these results suggest that 1,3-diCQA could modulate NO production by regulating nNOS expression and the ERK–CREB–BDNF signaling network.

### 3.5. 1,3-diCQA Directly Regulates nNOS Expression without Affecting Estrogen Receptors

**Figure 6:**
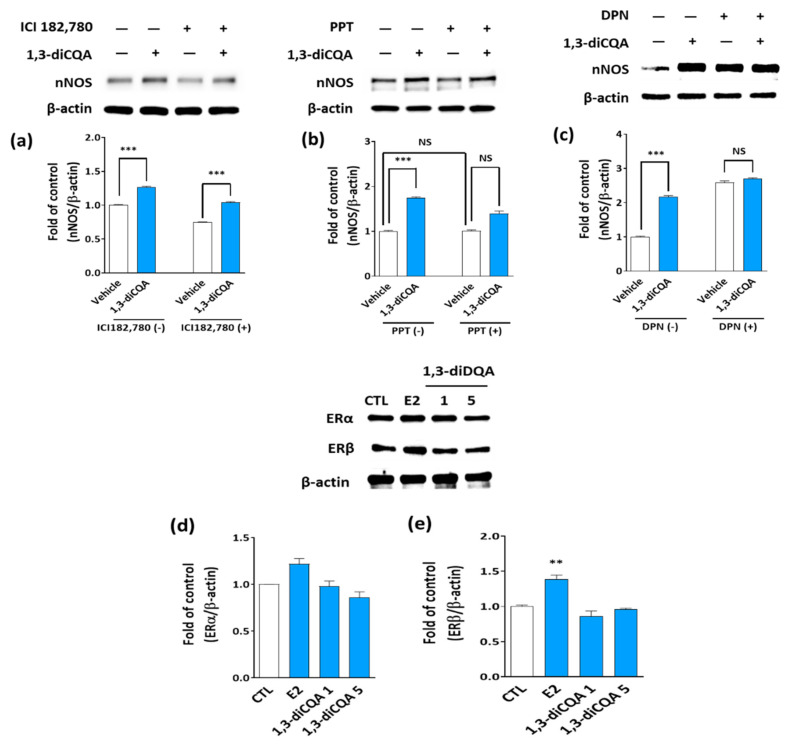
1,3-diCQA regulates nNOS expression directly rather than indirectly through ERs. Immunoblotting shows nNOS protein levels in the cultured SH-SY5Y cells treated by 5 uM 1,3-diCQA, with or without (**a**) 1 μM ICI180,720 (a nonselective ERs antagonist), (**b**) 10 μM PPT, or (**c**) 10 μM DPN, for 24 h. (**d**,**e**) 1,3-diCQA treatment did not affect ERs in the primary neuronal cells. Results are presented as mean ± SD. ** *p* < 0.01, *** *p* < 0.001 vs. vehicle-only treated cells, NS; not significant.

We determined whether 1,3-diCQA could affect estrogen receptor α (ERα) and estrogen receptor β (ERβ) in a similar manner to E2 using an ER agonist and antagonist in SH-SY5Y cells. The results show that nNOS levels were promoted following incubation of SH-SY5Y cells with 1,3-diCQA; meanwhile, the addition of ICI182,780 (an ERs antagonist), PPT (an ERα agonist), or DPN (an ERβ agonist) did not affect the stimulatory effect of 1,3-diCQA on nNOS (Figure 6a–c), which was consistent with the findings obtained in the primary neuronal cells (Figure 6d,e). These results suggest that 1,3-diCQA does not affect the ERs in the primary neuronal cells.

### 3.6. 7-NI Abolished the Behavioral Effects of 1,3-diCQA in OVX Mice

**Figure 7:**
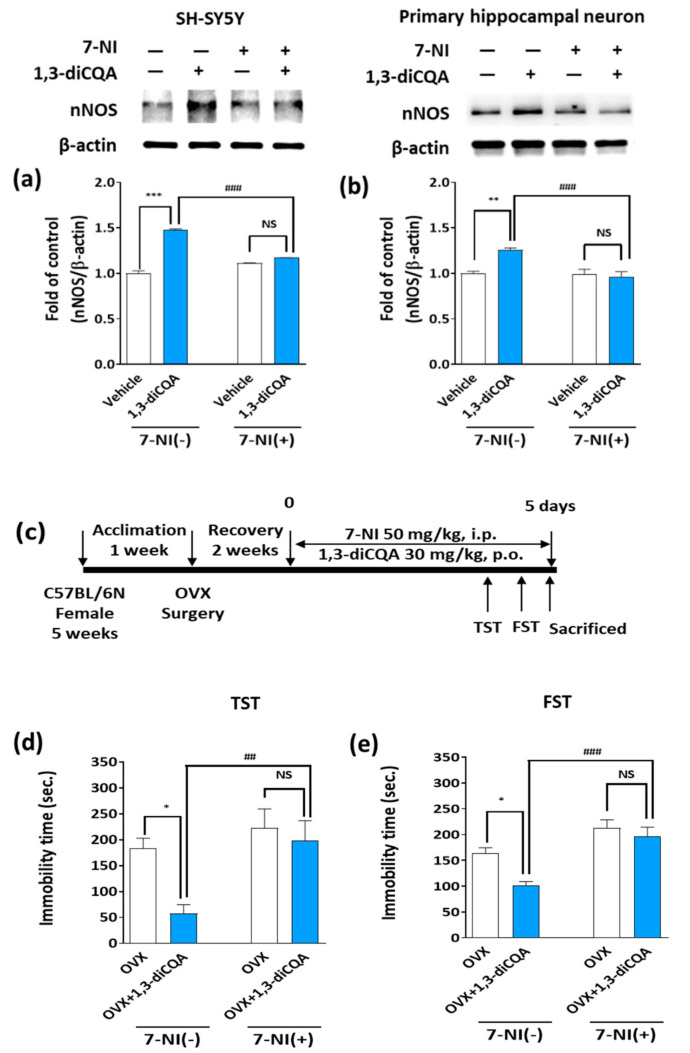
Effects of a specific nNOS inhibitor, 7-NI, on nNOS levels induced by 1,3-diCQA. SH-SY5Y human neuroblastoma cells and primary hippocampal neurons were incubated with 5 µM 1,3-diCQA for 24 h in the presence or absence of 7-NI (200 µM for 6 h). 1,3-diCQA induced nNOS level, whereas 7-NI inhibits this effect in the (**a**) SH-SY5Y cells and (**b**) primary hippocampal neurons. (**c**) Schematic illustration showing the experimental schedule. Anti-immobility effect of 1,3 diCQA is reversed by 7-NI co-treatment in (**d**) TST and (**e**) FST in OVX mice. Results are presented as mean ± SD. * *p* < 0.05, ** *p* < 0.01, *** *p* < 0.001 vs. OVX group; ## *p*  <  0.01, ### *p* < 0.001 vs. OVX+1,3-diCQA group, NS; not significant.

We first determined whether 1,3-diCQA directly regulates nNOS by treating SH-SY5Y cells and primary hippocampal neurons with 7-NI, a selective inhibitor of nNOS. Immunoblotting images show that nNOS protein levels in the SH-SY5Y cells exposed to 1,3-diCQA significantly increased; however, this effect was lost after 6 h of 7-NI treatment (Figure 7a). Similar results were observed in the primary hippocampal neurons (Figure 7b). To confirm the above results, we performed an in vivo study and observed that only OVX mice treated with 1,3-diCQA had significantly reduced immobility times in both the TST and FST, while pretreatment with 7-NI significantly reversed the antidepressant-like effect of 1,3-diCQA (Figure 7d,e). Thus, our findings demonstrate that 1,3-diCQA directly regulates nNOS, rather than indirect regulation via the ER, resulting in increased NO production.

## 4. Discussion

In the present study, we determined that AE and its main active component, 1,3-diCQA, elicited antidepressant-like effects on OVX-induced depressive-like behaviors via regulation of NO production by nNOS expression and the hippocampal ERK–CREB–BDNF signaling pathway in an ER-independent manner.

Estrogen deficiency may increase women’s vulnerability to depression, and estrogen therapy may be considered for the treatment of depressive symptoms during menopause [37]. These randomized controlled trial reports are consistent with preclinical evidence indicating that OVX significantly increases depressive-like behavior in rodents, including increased duration of immobility [38]. The state of immobility in the TST or FST is reported to mimic the depression phenotypes in humans and can be ameliorated by treatment with E2 [39]. As expected, in the present study, we observed a significant reduction in locomotor activity and increased immobility in OVX mice during the OFT, TST, and FST compared to the results for the SHAM mice. Meanwhile, AE treatment prevented this OVX-induced depressive-like behavior. Furthermore, the high dose of AE showed similar antidepressant effects to IMI and E2 in all behavioral tests. IMI is a tricyclic antidepressant that is clinically approved and commonly administered as an antidepressant. Our findings, therefore, demonstrate an antidepressant-like action of AE in a mouse model of depression induced by estrogen deficiency.

NO has been suggested to play a major role in the pathogenesis of depression [40]. Meanwhile, several studies have reported that NOS inhibitors have antidepressant-like properties [41]. However, Hu et al. [12] suggested that estrogen-mediated NO and nNOS expression in the hippocampus is critical for the observed sex differences in affective behaviors; specifically, NO shortage in the hippocampus contributes to OVX-induced depressive-like behaviors in mice. Similarly, in our study, OVX mice exhibited significantly lower hippocampal NO levels than the SHAM group, whereas AE or E2 administration reversed the reduction of hippocampal NO levels induced by estrogen deficiency. These results are consistent with a previous study that reported that certain functions of estrogen in the central nervous system are related to an increase in NO production [42]. Indeed, we observed an increase in nNOS mRNA and protein expression in the hippocampus and prefrontal cortex of AE-treated OVX mice. These results support the AE-induced number of nNOS-positive neurons in the immunofluorescence findings of the mice.

As previously reported, AE contains many phenolic compounds, including caffeoylquinic acid, in which esters of caffeic acid with quinic acid form derivatives, such as 1,3-diCQA, 3,4-diCQA, and 3,5-diCQA [18]. Here, we found that 1,3-diCQA as an active compound enhanced NO production by regulating nNOS expression in primary hippocampal neurons. These results suggest that AE and 1,3-diCQA exhibit antidepressant-like effects in OVX mice, which may be mediated via hippocampal NO production owing to nNOS upregulation.

CREB, a transcription factor necessary for neuronal survival, has been implicated in the signaling pathway associated with the pathogenesis of depression [43]. CREB is upregulated and activated, particularly in the hippocampus, by chronic antidepressant treatment in an animal study [44]. Moreover, the ERK pathway is activated by neurotrophins, and ERK has a potential role in the regulation of mood and depression-related behavior in human and animal studies [45]. The phosphorylated form of ERK leads directly to CREB phosphorylation, and enhanced CREB upregulates the expression of BDNF [46]. Meanwhile, a previous study has reported that reduced BDNF has been implicated in depression, and fluoxetine, a selective serotonin-uptake inhibitor, exerts antidepressant effects through the ERK-CREB signaling system [47]. BDNF, a neurotrophic factor, plays a major role in the neuronal system. According to the neurotrophin hypothesis of depression, BDNF and its receptor, TrkB, serve as important mediators in mental disorders, such as depression [48]. Specifically, BDNF mRNA levels are reportedly low in animal studies of depression or stress, while this reduction is ameliorated in animals administered antidepressants [49]. Similarly, low serum BDNF levels were reported in patients with major depressive disorder; however, the levels were elevated by antidepressant treatment [50]. As expected, our results showed that the phosphorylation of ERK, CREB, and the TrkB receptor, as well as the level of BDNF protein expression in the hippocampus, were significantly decreased in the OVX group compared with the SHAM group. However, these changes were significantly reversed following AE administration. In accordance with this, 1,3-diCQA consolidated the ERK–CREB–BDNF pathway in primary hippocampal neurons. Thus, our results suggest that AE prevents OVX-induced depression-like behavior through upregulation of the ERK–CREB–BDNF signaling pathway via its main phenolic compound, 1,3-diCQA.

To the best of our knowledge, ERα and ERβ are associated with the pathophysiology of menopause-related neurological and psychiatric disorders [51]. In rats, ERα knockdown in the posterodorsal amygdala of female mice reduced anxiety-like behavior, as demonstrated by the increased time spent in, and number of visits to, the light chamber of the light/dark box [52]. In addition, ERβ agonists decreased the immobility time of OVX rats in the FST [53]. Furthermore, ERβ activation in the CA3 region of the hippocampus induced by DPN slows serotonin clearance via the MAPK/ERK1/2 signaling pathway and its interactions with both TrkB receptors [54]. These findings suggest that activation of ERα and ERβ induces antidepressant effects in female rodents. For this reason, we investigated whether 1,3-diCQA mediates ERα and/or ERβ. Interestingly, 1,3-diCQA treatment did not affect ERα or ERβ expression levels in primary hippocampal neurons. Additionally, we found that 1,3-diCQA modulates NO production via nNOS upregulation without affecting the ERs. However, it is not surprising that our results differ from those of previous studies since the effect of 1,3-diCQA on hippocampal NO production, as we have shown, is not mediated by ERs. Our results indicate that the antidepressant effect of 1,3-diCQA may regulate other pathways.

**Figure 8:**
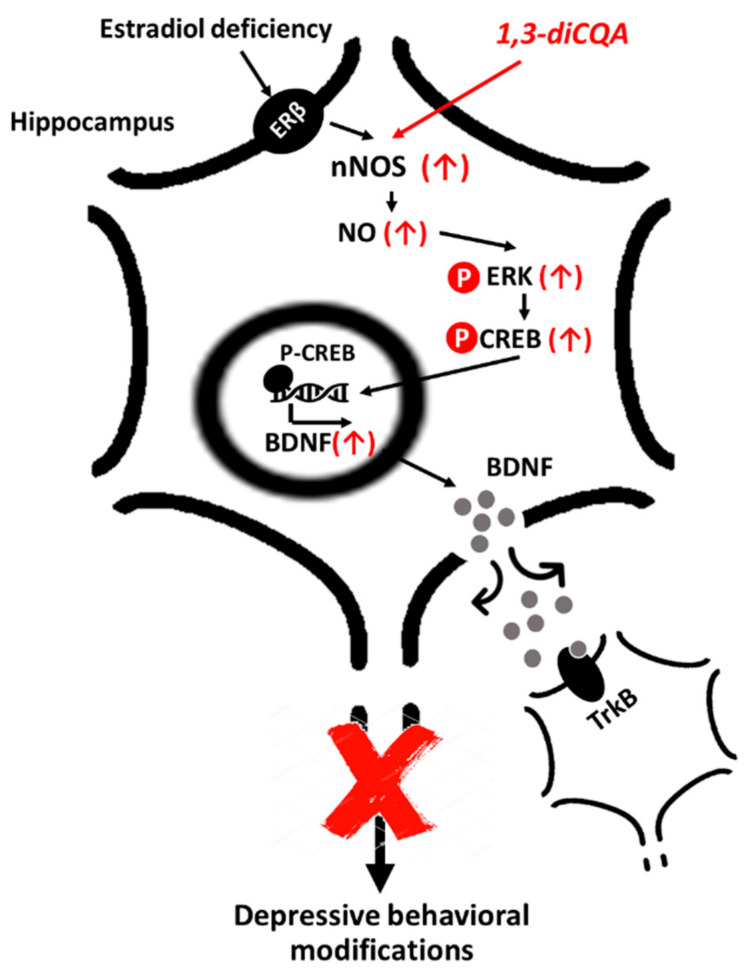
Schematic illustrating the mechanism underlying the effect of 1,3-diCQA in the hippocampal neuron. 1,3-diCQA promotes NO production via upregulating nNOS expression without affecting the ERs. 1,3-diCQA may induce changes in depression-like behaviors through the ERK–CREB–BDNF signaling pathway in the hippocampus. Thus, 1,3-diCQA prevents estrogen-deficiency-induced depressive behavioral modifications through the nNOS-NO/ERK–CREB–BDNF signaling pathway network. nNOS; neuronal nitric oxide synthase, NO; nitric oxide, ERK; extracellular-signal-regulated kinases, CREB; cyclic-AMP response-element-binding protein, TrkB: tropomyosin receptor kinase B, ERβ; estrogen receptor beta, 1,3-diCQA; 1, 3-dicaffeoylquinic acid.

To confirm the participation of the nNOS signaling pathway in the antidepressant effect of 1,3-diCQA, we next examined the influence of nNOS inhibitors on in vitro and in vivo models. We found that 1,3-diCQA induced nNOS expression in SH-SY5Y human neuroblastoma cells and primary hippocampal neurons, while this alteration was completely abolished by co-treatment with 7-NI. Moreover, we confirmed that 7-NI could abolish the antidepressant-like effect of 1,3-diCQA treated OVX mice in the TST and FST test, demonstrating that the activation of nNOS plays a critical role in its antidepressant effect. Likewise, Hu et al. [12] showed that the basal level of hippocampal NO in the female hippocampus is lower than that found in male mice; meanwhile, 7-NI significantly increased the immobility times of OVX mice in the TST and FST, whereas immobility times of NO donor DETA/NONOate-treated OVX mice increased, thus mimicking the effects of antidepressants. Moreover, Zhang et al. [34] reported that levels of nNOS, NO, and p-CREB were increased in E2- and progesterone-treated OVX mice, followed by a significant decline in early E2 withdrawal mice; whereas 7-NI or C-PTIO (NO scavenger) treatment caused depressive-like behavior and reduced hippocampal p-CREB in E2- and progesterone-treated OVX mice. Therefore, our findings indicate that 1,3-diCQA can alleviate depression-like phenotypes through stimulation of hippocampal nNOS-NO activities without the involvement of ERs (Figure 8).

A major strength of this study is that it provides a possible explanation for the mechanism by which 1,3-diCQA or AE could ameliorate depression-like behavior induced by OVX. However, there are some limitations to this study: the change in plasma levels of estrogen after ovariectomy does not necessarily reflect the steroid levels in either the peripheral or central nervous system in rodents [55], and the OVX model also does not fully reflect menopausal symptoms by natural reproductive senescence [56]. Therefore, the antidepressant efficacy of 1,3-diCQA should be considered in various animal models of menopause, such as natural reproductive senescence [57], and ovotoxin-induced ovarian failure [58] in future studies, including clinical trials.

## 5. Conclusions

The results of this study support our hypothesis that 1,3-diCQA may prevent depression-like behaviors in estrogen deficiency-induced depression by increasing hippocampal NO formation. This beneficial effect may be mediated by direct regulation of nNOS expression and ERK–CREB–BDNF signaling networks, however, not by mediating ERs. Taken together, our findings indicate that 1,3-diCQA may serve as a potent therapeutic candidate for treating menopausal depression by regulating nNOS and the ERK–CREB–BDNF signaling network.

## Figures and Tables

**Table 1:** Antibodies used in this study.

Antibody	Source	Identifier	Dilution
Mouse monoclonal anti-β-actin	Santa Cruz	Cat# sc-47778	1:1000
Rabbit monoclonal anti-CREB	Cell signaling technology	Cat# 9197S	1:1000
Rabbit monoclonal anti-p-CREB	Cell signaling technology	Cat# 9198S	1:1000
Rabbit monoclonal anti-p44/42 MAPK (Erk1/2)	Cell signaling technology	Cat# 4695S	1:1000
Rabbit monoclonal anti-p-p44/42 MAPK (Erk1/2) (Thr202/Tyr204)	Cell signaling technology	Cat# 4377S	1:1000
Rabbit monoclonal anti-TrkB	Cell signaling technology	Cat# 4603S	1:1000
Rabbit polyclonal anti-p-TrkB (Tyr816)	Millipore	Cat# ABN1381	1:500
Rabbit monoclonal anti-nNOS	Abcam	Cat# ab76067	1:1000
Rabbit polyclonal anti-ERβ	Abcam	Cat# ab3567	1:1000
Rabbit monoclonal anti-BDNF	Abcam	Cat# ab108319	1:1000
Anti-Neurofilament heavy polypeptide antibody	Abcam	Cat# ab4680	1:1000
Alexa 488-conjugated Goat anti-Rabbit IgG	Abcam	Cat# ab150077	1:200
Alexa 555-conjugated Goat anti-Chicken IgG	Abcam	Cat# ab150170	1:200
Goat Anti-Rabbit IgG Antibody (H+L), Peroxidase	Vector laboratories	Cat# PI-1000	1:5000

## Data Availability

The data presented in this study are available on request from the corresponding authors.
